# Age-Related Alterations in Human Gut Microbiota and Intestinal Aging: Microbial Signatures, Disease Associations and Intervention Strategies

**DOI:** 10.3390/ijms272110043

**Published:** 2026-11-09

**Authors:** Siyu Li, Minjia Chen, Huan Yang, Xuefei Zhao, Yuting Tian, Yi Cheng, Qiwei Wu, Yangyang Feng, Lu Chen, Ailin Yi, Guangcong Ruan, Yanling Wei

**Affiliations:** Chongqing Key Laboratory of Digestive Malignancies, Department of Gastroenterology, Engineering Research Center for Advanced Medical Technology in Microecological Therapy of Chongqing, Daping Hospital, Army Medical University (Third Military Medical University), Chongqing 400042, China; lsylsy@tmmu.edu.cn (S.L.); chenmj525@tmmu.edu.cn (M.C.); yanghhh2122@163.com (H.Y.); xfzhao20@tmmu.edu.cn (X.Z.); tianyuting@tmmu.edu.cn (Y.T.); chengyidph@tmmu.edu.cn (Y.C.); wqw123456@tmmu.edu.cn (Q.W.); youngyangfeng@tmmu.edu.cn (Y.F.); chenlu130@tmmu.edu.cn (L.C.); yalyal120@tmmu.edu.cn (A.Y.)

**Keywords:** aging, gut microbiota, intestinal aging, age-related diseases, microbiota-targeted intervention

## Abstract

Aging is accompanied by progressive alterations in gut homeostasis, characterized by gut microbiota dysbiosis, impaired intestinal barrier function, immune dysregulation, and chronic low-grade inflammation. Increasing evidence indicates that age-related gut microbial remodeling is closely associated with the development of multiple age-associated disorders, including frailty, sarcopenia, cardiovascular diseases, and neurodegenerative diseases. However, existing reviews have primarily focused on individual aspects of gut microbiota-mediated aging, whereas a systematic framework integrating gut microbial dysbiosis, intestinal aging, and the development of multiple age-related diseases through shared pathogenic mechanisms remains lacking. This review provides a comprehensive synthesis of age-related alterations in human gut microbiota, with particular emphasis on bacterial taxa consistently associated with healthy aging and age-related diseases. We further integrate current evidence to examine the associations between age-related microbial alterations and intestinal aging, with particular attention to microbial function, barrier integrity, and host immune responses. Finally, we review microbiota-targeted interventions, including fecal microbiota transplantation, probiotics, prebiotics, and dietary modulation, and discuss their potential and limitations in promoting healthy aging. By integrating mechanistic evidence with findings from human observational cohorts and clinical intervention studies, this review provides a translational framework for understanding microbiota-mediated intestinal aging and highlights future directions for precision microbiota-based strategies to promote healthy aging.

## 1. Introduction

Aging is a complex biological process characterized by progressive functional decline across multiple organs and increased susceptibility to a broad range of age-associated conditions [1,2]. Although these conditions affect different physiological systems, they share several features associated with aging, including chronic low-grade inflammation, impaired tissue homeostasis, immune dysregulation, and metabolic alterations. Increasing evidence suggests that the gut microbiota may be involved in these processes through interactions with multiple organs and systems, including the musculoskeletal, cardiovascular, and nervous systems [3,4,5]. At the molecular and cellular levels, biological aging is characterized by multiple interconnected hallmarks, including epigenetic alterations, loss of proteostasis, altered intercellular communication, chronic inflammation, and gut microbial dysbiosis [6].

The gastrointestinal tract is one of the organs most profoundly affected by aging. Age-related intestinal dysfunction is characterized by alterations in gut microbiota composition, impaired intestinal barrier integrity, and reduced physiological function, which are associated with both local intestinal disorders and systemic diseases [7,8,9]. The gut microbiota plays a critical role in maintaining host homeostasis by regulating nutrient metabolism, immune responses, epithelial integrity, and microbe–host interactions. Aging is accompanied by progressive remodeling of the gut microbial ecosystem, including alterations in microbial composition and functional capacity. These age-related microbial alterations may influence intestinal and systemic homeostasis through interconnected metabolic, barrier, and immune pathways [3].

Recent advances have highlighted the potential role of the gut microbiota not only as a biomarker but also as a therapeutic target in intestinal aging [10]. Although the gut microbiota has been widely implicated in aging, existing reviews have often addressed specific aspects of this process, including age-related microbial alterations, intestinal barrier and immune dysfunction, longevity, or microbiota-targeted interventions [11]. However, an integrated framework linking gut microbial dysbiosis and intestinal aging with the shared biological features across multiple age-related diseases remains insufficiently developed. In particular, microbial features shared across age-associated conditions are not always clearly distinguished from condition-specific signatures, taxonomic associations are frequently discussed together with functional or metabolomic findings despite representing different levels of evidence, and mechanistic observations from experimental models are not always clearly separated from demonstrated effects in humans. These limitations complicate assessment of the biological specificity and translational relevance of microbiota–aging associations. Furthermore, although accumulating human cohort and intervention studies have expanded the clinical evidence base, mechanistic evidence derived from experimental models remains prominent in the field, highlighting the need for greater integration of human evidence when evaluating the translational relevance of microbiota-mediated intestinal aging [12].

To address these gaps, this review adopts a comparative and evidence-oriented framework for evaluating age-related alterations in the human gut microbiota and intestinal aging. We focus on representative conditions spanning systemic and multisystem functional decline and distinct gut–organ interactions, including the gut–muscle, gut–cardiovascular, and gut–brain axes. Across these conditions, we distinguish shared from condition-specific microbial features and evaluate taxonomic associations separately from functional, metabolomic, and mechanistic evidence. We further consider the extent to which findings are supported by human observational studies, experimental models, or clinical interventions when assessing their translational relevance. Within this framework, we discuss intestinal barrier function, immune regulation, microbial metabolism, and microbiota-targeted interventions, and identify key limitations and priorities for future studies of microbiota-mediated healthy aging.

To support this, relevant literature was searched in PubMed between September 2025 and September 2026, with the final search conducted in September 2026. The search used combinations of the terms “aging”, “gut microbiota”, “intestinal aging”, “age-related diseases”, and “microbiota-targeted intervention”. Studies were considered if they addressed age-associated changes in the gut microbiota, intestinal aging, microbiota alterations in age-related diseases, or microbiota-targeted interventions in the context of aging. Original human observational studies and clinical trials were prioritized when assessing associations and intervention outcomes in humans, whereas animal and other experimental studies were included to evaluate potential mechanisms. Reviews and meta-analyses were used mainly to provide background context and to identify relevant primary studies. The selected literature was organized according to the major themes of this review, and findings were compared across studies with particular attention to consistency and discrepancies, differences in study populations and experimental settings, and distinctions between human and preclinical evidence.

## 2. Changes in General Features During Aging

The gut microbiota serves as an interface between the external environment and the host. Gut microorganisms begin colonizing the gastrointestinal tract after birth, and the gut microbial community becomes increasingly established during early childhood. The structure and diversity of the gut microbiota are closely related to lifestyle, geographical environment, dietary habits, ethnicity, and other factors. The gut microbiota forms a mutualistic relationship with the host, playing crucial physiological roles by promoting nutrient digestion, regulating immune responses, and maintaining intestinal homeostasis. Among these, the four bacterial phyla *Firmicutes*, *Bacteroidetes*, *Proteobacteria*, and *Actinobacteria* account for approximately 98% of the total microbial population [13,14].

Numerous human cohort studies, including cross-sectional analyses, longitudinal investigations, and population-based studies conducted in different countries, consistently demonstrate that aging is accompanied by profound alterations in gut microbial composition and function. These changes occur in the context of multiple age-related factors, including altered dietary habits, impaired digestive and absorptive capacity, reduced physical activity, frequent hospitalization, increased susceptibility to infections, and extensive antibiotic exposure. Collectively, these factors are associated with age-related alterations in gut microbial composition and function, although the direction and magnitude of these changes vary across populations [9,14,15,16]. In humans, several studies have reported an age-associated decline in gut microbial diversity. Alpha diversity has been reported to decrease in older adults (excluding centenarians), whereas beta diversity may remain relatively stable between younger and older populations [15].

Specifically, overall gut microbial diversity has been reported to decline in older adults, accompanied by reduced abundance of multiple bacterial species, including *Prevotella copri*, *Coprococcus eutactus*, *Faecalibacterium prausnitzii*, *Roseburia inulinivorans*, *Roseburia intestinalis*, *Eubacterium hallii*, *Barnesiella intestinihominis*, *Alistipes putredinis*, *Parabacteroides merdae* and *Megamonas funiformis*. Notably, several of these taxa, including *F. prausnitzii*, *R. inulinivorans*, *Roseburia intestinalis*, and *E. hallii*, have been associated with SCFA (short-chain fatty acid)-related functions [17,18]. Conversely, increased abundances of several taxa, including *Klebsiella pneumoniae*, *Escherichia coli*, *Clostridium hathewayi*, *Clostridium bolteae*, *Bacteroides fragilis*, *Veillonella unclassified*, *Escherichia unclassified*, and *Mitsuokella multacida*, have been reported and are associated with various health risks [15,17,18,19,20,21,22,23,24,25,26]. Collectively, evidence from multiple human populations indicates substantial age-associated remodeling of the gut microbiota, although the specific taxonomic patterns are not fully consistent across studies. More detailed age-specific microbial profiles are summarized in Table 1.

Compared with non-centenarian older adults, centenarians have been reported to exhibit greater microbial diversity and distinct microbial community structures [26,27,28,29]. *Escherichia*, *Clostridium*, *Pseudomonas*, *Bacteroides*, *Prevotella*, *Sutterella*, *Roseburia* and *Butyricicoccus* levels were lower in centenarians, whereas *Faecalibacterium*, *Bifidobacterium*, *Prevotella*, *Anaerotruncus*, *Akkermansia*, *Bilophila*, *Christensenellaceae*, *Parabacteroides*, *Butyricimonas* and *Alistipes* levels were higher [9,17,22,23,26,30,31]. Notably, findings for *Prevotella* have been inconsistent across studies, with both lower and higher relative abundances reported in centenarians compared with their respective reference groups. These findings suggest that gut microbial features associated with healthy longevity are population-dependent rather than universally conserved. Therefore, microbiota-targeted interventions should increasingly consider age-specific microbial characteristics, shifting from generalized probiotic strategies toward precision microbiome modulation based on the features of gut microbiota.

Biological sex appears to modify age-associated changes in the gut microbiota. Compared with men, premenopausal women exhibited higher microbial diversity and greater abundances of several species associated with favorable host metabolic features, while these sex-related differences generally diminished with advancing age [32]. In adults aged 51–89 years, microbial aging trajectories also differed by sex, with a greater decline in microbial richness in women and a stronger age-related increase in community uniqueness in men; most aging-associated microbial guilds were also sex-specific [33]. Sex-related differences persisted in centenarians at both taxonomic and functional levels, including microbial functions related to oxidative-stress resistance [34]. Collectively, these findings indicate that biological sex modifies gut microbial trajectories across aging, although some age-associated features are shared between sexes.

Animal studies generally support these observations while providing mechanistic insights into age-associated microbial remodeling. Comparative analyses between young and aged mice have demonstrated significant differences in microbial composition; however, the specific taxa reported vary across studies. One investigation found increased abundances of *Lactobacillus murinus*, *Lactobacillus reuteri*, *Bacteroides acidifaciens*, *Akkermansia muciniphila*, *Coriobacteriaceae* UCG-002, *Erysipelotrichaceae bacterium*, *Lactobacillus johnsonii*, and *Ileibacterium valens* in aged mice, whereas members of the *Lachnospiraceae* NK4A136 group, *Dubosiella*, *Clostridiales* bacterium, and *Bifidobacterium pseudolongum* were enriched in young animals [8]. Another independent study reported reduced abundances of *Akkermansia muciniphila*, *Porphyromonas*, *Collinsella*, *Corynebacterium*, and *Lactobacillus gasseri*, together with increased *Lachnospira pectinoschiza* and *Butyrivibrio crossotus* in aged mice [35]. These inconsistencies likely reflect differences in mouse strains, housing environments, dietary conditions, sequencing platforms, and analytical pipelines. Nevertheless, animal models consistently demonstrate that aging induces substantial gut microbial remodeling and provides valuable mechanistic evidence complementing observations from human studies. Therefore, when identifying therapeutic probiotics, personalized considerations, such as age, should be incorporated, shifting research from generic probiotics toward personalized strains. Additionally, the microbiomes of centenarians differ from those of non-centenarian seniors, and their longevity-associated features warrant attention. Future research should focus on the microbiota of healthy, long-lived elderly individuals to identify methods for improving age-related physiological impairments.

Overall, the studies summarized in Table 1 suggest that gut microbial composition changes with aging, although the direction and magnitude of these changes are not entirely consistent across populations. For example, age-associated decreases in taxa such as *Faecalibacterium* and *Roseburia* have been reported in some studies, while *Akkermansia*, *Alistipes*, and *Barnesiella* have been associated with exceptional longevity in specific populations. These differences may reflect variations in study populations, age ranges, dietary patterns, geographic backgrounds, taxonomic resolution, and analytical methods. Therefore, the identified taxa should be interpreted as population- and study-dependent microbial signatures rather than universal biomarkers of aging.

**Table 1:** Representative gut microbial taxa associated with aging and longevity in human studies.

Age Range (Years)	Taxonomic Level	Representative Microbial Taxa	Direction	Comparison/Analysis
5–80	Genus	*Erysipelatoclostridium*, *Bifidobacterium*	↓	Age-associated trend [24]
16–108	Genus	*Odoribacter*, *Desulfovibrio*, *Ruminococcaceae* UCG-003	↑	Age-associated trend [26]
16–108	Species	*Desulfovibrio piger*, *Gordonibacter pamelaeae*, *Odoribacter splanchnicus*, *Ruminococcaceae bacterium* D5	↑	Age-associated trend [26]
18–107	Species	*Finegoldia magna*, *Bifidobacterium dentium*, *Clostridium clostridioforme*	↑	Age-associated trend [20]
18–107	Species	*Prevotella copri*	↑	Age-associated trend [20]
21–100	Genus	*Bacteroides*	↓	Age-associated trend [18]
21–100	Genus	*Parasutterella*	↓	Age-associated trend [18]
21–100	Species	*Alistipes shahii*, *Alistipes onderdonkii*, *Alistipes senegalensis*, *Bacteroides xylanisolvens*, *Bacteroides dorei*	↑	Age-associated trend [18]
21–100	Species	*Faecalibacterium prausnitzii*, *Roseburia inulinivorans*	↓	Age-associated trend [18]
≥50	Species	*Bacteroides fragilis*, *Bifidobacterium longum*, *Clostridium bolteae*, *Escherichia coli*, *Klebsiella pneumoniae*, *Parabacteroides merdae*	↑	Other age groups (<50 years) [25]
≥50	Species	*Barnesiella intestinihominis*, *Megamonas funiformis*	↓	Other age groups (<50 years) [25]
≥60	Genus	*Coprococcus*	↑	Other age groups (50–59 years old) [21]
90–99	Genus	*Coprococcus_2*	↑	Other age groups (65–70 years old) [31]
≥90	Species	*Bacteroides fragilis*, *Parabacteroides merdae*, *Ruminococcus gnavus*, *Clostridium perfringens*	↑	Other age groups (65–70 years old) [31]
≥90	Species	*Bacteroides vulgatus*	↓	Other age groups (65–70 years old) [31]
>90	Genus	*Akkermansia*	↑	Other age groups (24–83 years) [22]
95–108	Genus	*Faecalibacterium*, *Prevotella*	↓	Other age groups (26–69 years) [30]
95–108	Genus	*Escherichia*, *Akkermansia*, *Clostridium*, *Collinsella*	↑	Other age groups (26–69 years) [30]
>100	Genus	*Alistipes*, *Barnesiella*	↑	Other age groups (65–70 years) [31]
>100	Genus	*Barnesiella*, *Butyricimonas*, *Butyricicoccus*	↑	Other age groups (90–99 years) [31]
100–108	Genus	*Parabacteroides*, *Butyricimonas*, *Alistipes*	↑	Other age groups (16–83 years) [26]
100–108	Genus	*Dialister*, *Parasutterella*, *Acidaminococcus*	↓	Other age groups (16–83 years) [26]
105–109	Genus	*Akkermansia*, *Eggerthella*, *Anaerotruncus*, *Bilophila*, *Oscillospira*	↑	Other age groups (22–104 years) [23]
105–109	Genus	*Faecalibacterium*, *Roseburia*, *Coprococcus*	↓	Other age groups (22–104 years) [23]

Note: Age ranges and comparison groups were defined according to the original studies. For direct age-group comparisons, upward (↑) and downward (↓) arrows indicate higher and lower microbial relative abundances, respectively, in the SASP indicated age group compared with the reference group. For studies reporting age-associated trajectories or correlations, arrows indicate increasing or decreasing microbial abundance with age rather than direct differences between predefined age groups. For logistic regression analyses, arrows indicate positive or negative associations with longevity-group membership rather than direct abundance differences.

## 3. Gut Microbiota in Representative Age-Associated Conditions

Given the broad spectrum of age-associated conditions linked to the gut microbiota, representative conditions were selected based on their relevance to aging, the availability of human gut microbiota evidence, and their representation of distinct gut–organ interactions. Frailty was included to represent systemic and multisystem functional decline; sarcopenia to represent the gut–muscle axis; cardiovascular disease to represent the gut–cardiovascular axis; and Parkinson’s disease (PD) and Alzheimer’s disease (AD) to represent the gut–brain axis. Including both PD and AD allows comparison of shared and disease-specific microbiota-related features within the same gut–organ axis. This framework therefore enables comparison of both shared and condition-specific microbiota-related features across and within gut–organ axes. Representative microbial alterations, functional findings, and the corresponding levels and limitations of current evidence are summarized in Table 2.

### 3.1. Frailty

Recent population-based studies have identified gut microbial alterations associated with frailty in older adults. However, frailty-associated microbial changes are primarily characterized by alterations in microbial composition rather than a consistent decline in overall diversity. A systematic review and meta-analysis of 11 observational studies by Almeida et al. identified frailty-associated alterations in several commensal and potentially opportunistic taxa [36].

Several frailty-associated studies have reported reduced abundances of taxa with known or proposed SCFA-related functions, including *Faecalibacterium*, *Roseburia*, *Coprococcus*, and *Blautia*. At the species level, *Faecalibacterium prausnitzii* is a well-characterized butyrate-producing bacterium and has been reported to be reduced in frailty. In contrast, potentially pro-inflammatory and opportunistic bacteria, such as *Alistipes*, *Parabacteroides*, *Enterobacteriaceae*, *Escherichia coli*, *Desulfovibrio*, and *Klebsiella*, are enriched in frail older adults [36,37,38,39]. These compositional alterations indicate a transition from a homeostatic microbiota toward a pro-inflammatory microbial ecosystem [38].

Beyond taxonomic alterations, these compositional alterations are accompanied by profound functional consequences. Several cohort studies have demonstrated significant associations between specific microbial taxa and clinical manifestations of frailty, including reduced grip strength, impaired physical performance, appetite decline, and muscle dysfunction [39,57], supporting the emerging concept of the gut–muscle axis in frailty development. These observations have led to the hypothesis that microbial metabolic outputs, rather than taxonomic composition alone, may be relevant to frailty-associated physiological changes. Alterations in taxa associated with SCFA-related and other metabolic functions have been reported in frailty; however, these taxonomic changes do not necessarily indicate corresponding changes in metabolite production. Functional and metabolomic studies have also implicated SCFAs, bile acids, and aromatic amino acid-derived metabolites in pathways related to immune regulation, mitochondrial function, skeletal muscle metabolism, and cellular senescence. However, the extent to which these pathways causally contribute to frailty in humans remains uncertain. Collectively, these findings highlight a close relationship between gut microbial alterations and the clinical and metabolic features of frailty.

### 3.2. Sarcopenia

Accumulating evidence indicates that sarcopenia is associated with alterations in gut microbial composition and functional potential. Although findings vary across study populations, geographic regions, dietary patterns, diagnostic criteria, and sequencing methods, several recurrent microbial alterations have been reported [40,41]. Systematic reviews have identified recurrent alterations in several taxa, including reduced abundances of *Faecalibacterium*, *Prevotella*, and *Blautia*, although substantial between-study heterogeneity remains. In a study of community-dwelling older adults using 16S rRNA sequencing, Lee et al. reported lower abundances of *Prevotella* and *Prevotella copri* and a higher abundance of *Parabacteroides* sp. in participants with sarcopenia than in those without sarcopenia [42]. At the species level, in a population-based shotgun metagenomic study of 1417 participants, *Desulfovibrio piger*, *Clostridium symbiosum*, *Hungatella effluvii*, *Bacteroides fluxus*, *Absiella innocuum*, *Coprobacter secundus*, and *Clostridium citroniae* were enriched in participants with sarcopenia after adjustment for potential confounders [43]. Six of these species were also positively associated with sarcopenia severity [43].

Beyond taxonomic composition, emerging evidence suggests that sarcopenia is also associated with alterations in microbial functional potential. Several microbial taxa, including *Faecalibacterium*, *Roseburia*, and *Prevotella*, have been reported to be positively associated with skeletal muscle mass, handgrip strength, gait speed, and Short Physical Performance Battery scores [44,58]. Shotgun metagenomic functional profiling revealed depletion of pathways related to phenylalanine, tyrosine, and tryptophan biosynthesis, together with enrichment of pathways related to α-linolenic acid metabolism, furfural degradation, and staurosporine biosynthesis [43].

Collectively, current human evidence supports an association between sarcopenia and alterations in gut microbial composition and functional potential. However, taxonomic findings remain heterogeneous across studies, and the predominantly observational nature of the available evidence precludes conclusions regarding a causal role of the gut microbiota in sarcopenia.

### 3.3. Cardiovascular Diseases

Aging is accompanied by progressive remodeling of gut microbiota composition and function. In a human study involving female participants, higher gut microbial α-diversity has been associated with lower arterial stiffness, suggesting a relationship between microbial diversity and vascular health during aging [59]. In contrast, patients with coronary artery disease exhibit decreased microbial richness and diversity, accompanied by depletion of potential SCFA-producing bacteria, including members of the Bacteroidetes phylum and Lachnospiraceae family [60]. At the broader evidence level, a systematic review and meta-analysis reported enrichment of Proteobacteria and *Streptococcus* and depletion of *Faecalibacterium* across cardiovascular disease studies, although substantial heterogeneity was observed among disease subtypes and study populations [45].

Beyond taxonomic composition, large-scale human multi-omic studies have identified associations between gut microbial metabolic functions and cardiovascular risk-related phenotypes [46]. A study integrating shotgun metagenomic and metabolomic profiling of 1429 participants identified microbial pathways related to cholesterol, flavonoid, and γ-butyrobetaine metabolism, with several *Oscillibacter* species associated with lower fecal and plasma cholesterol levels [47]. In addition, trimethylamine N-oxide (TMAO), a gut microbiota–host co-metabolite, represents a key mediator linking gut dysbiosis with vascular aging. Gut bacteria metabolize dietary precursors, such as choline, to produce trimethylamine, which is subsequently absorbed and oxidized to TMAO in the liver [61]. Circulating TMAO levels have been reported to increase with age and are associated with reduced flow-mediated dilation in humans. Experimental studies demonstrate that TMAO promotes oxidative stress, decreases nitric oxide bioavailability, and impairs endothelial function, whereas inhibition of microbiota-dependent trimethylamine production improves vascular function in aged mice [48].

Collectively, current evidence links cardiovascular phenotypes to both gut microbial composition and microbiota-associated metabolic functions. However, taxonomic findings vary across cardiovascular disease subtypes and cohorts, and human evidence for specific microbiota-derived metabolites remains predominantly associative, whereas direct mechanistic support is derived largely from experimental models.

### 3.4. Parkinson’s Disease

Parkinson’s disease (PD) is a common age-related neurodegenerative disorder. Increasing evidence indicates that PD is associated with alterations in gut microbial composition, although the reported taxonomic patterns vary across populations and studies [49,50]. Recurrent alterations include decreased abundances of Lachnospiraceae and Prevotellaceae and increased abundances of Verrucomicrobiaceae and Lactobacillaceae [49].

Beyond taxonomic alterations, human studies have identified changes in both microbial functional potential and metabolite profiles in PD. A large-scale shotgun metagenomic study involving 490 patients with PD and 234 controls identified extensive alterations in microbial species, genes, and metabolic pathways in PD [51]. Direct metabolite measurements showed lower fecal but higher plasma concentrations of acetate, propionate, and butyrate in patients with PD than in controls. Lower fecal acetate, propionate, and butyrate levels were also associated with greater motor impairment in patients with PD [52]. Meanwhile, expansion of certain taxa may increase oxidative stress, facilitate abnormal α-synuclein aggregation, and activate microglia. These processes collectively exacerbate neuroinflammation and accelerate dopaminergic neuronal injury [49,50].

However, substantial between-study heterogeneity remains, and most human evidence is observational; therefore, the causal contribution of gut microbiota alterations to PD pathogenesis remains to be established [49,50].

### 3.5. Alzheimer’s Disease

Alzheimer’s disease (AD) is an age-associated neurodegenerative disorder characterized by progressive cognitive decline and pathological changes in the brain. Increasing evidence indicates that gut microbiota alterations occur in patients with AD and may already be detectable during the stage of mild cognitive impairment, with additional changes during disease progression [53]. Across studies, AD-associated microbial alterations have included changes in microbial diversity and the relative abundances of several taxa, including several genera, such as *Faecalibacterium*, *Roseburia*, *Ruminococcus*, *Dialister*, *Lachnoclostridium*, *Eubacterium*, *Escherichia/Shigella*, and *Phascolarctobacterium*, as well as the species *Akkermansia muciniphila*, although the direction and consistency of these alterations vary across cohorts [54,55,56].

Beyond taxonomic alterations, emerging human evidence suggests that AD is also associated with changes in microbial functional potential. A large-scale human study identified stage-specific alterations in microbial gene families across the AD continuum, involving pathways related to carbohydrate, amino-acid, energy, glycan, and vitamin metabolism [53]. Another human shotgun metagenomic study further showed that microbial compositional and functional features were associated with cerebrospinal fluid (CSF) biomarkers of AD and related pathologies [62]. Experimental studies have linked microbiota-associated inflammatory signaling to amyloid-β (Aβ) deposition, Tau hyperphosphorylation, oxidative stress, and microglial activation [54,63]. In addition, increased abundance of *Bacteroides* may contribute to the activation of C/EBPβ signaling in microglia and C/EBPβ–AEP signaling in neurons, thereby promoting neuroinflammation and exacerbating AD pathology [63].

Collectively, current human evidence supports associations between gut microbial compositional and functional alterations and AD-related pathological features, whereas direct mechanistic evidence is derived primarily from experimental studies.

Overall, Table 2 highlights both shared and condition-specific patterns in gut microbial composition and function across the selected age-associated conditions. Several conditions show alterations in taxa associated with SCFA-related functions; however, taxonomic changes should not be interpreted as direct evidence of altered SCFA concentration. At the same time, disease- and cohort-specific differences are evident at both the taxonomic and functional levels, and no single microbial signature appears to be consistently shared across all conditions. These variations may reflect differences in study populations, disease stage or severity, diet, medication use, and analytical methods. Furthermore, the strength of evidence varies substantially, ranging from taxonomic associations and metagenomic functional potential to direct metabolite measurements and experimental mechanistic evidence. Most human evidence remains observational, limiting causal interpretation. Longitudinal, multi-omic, and interventional studies are therefore needed to determine which microbiota-related features precede disease progression and which have causal or translational relevance.

## 4. Intestinal Barrier and Functional Changes During Aging

### 4.1. Changes in the Intestinal Barrier

During aging, alterations in the gut microbiota are closely associated with impaired intestinal barrier integrity and chronic low-grade inflammation, potentially increasing susceptibility to gastrointestinal infections such as *Clostridium difficile* and *Helicobacter pylori* [9,23]. Age-related loss of barrier integrity is associated with alterations in metabolic and immune signaling, and intestinal barrier dysfunction is a stronger predictor of age-related mortality than chronological age [64].

#### 4.1.1. Changes in Intestinal Permeability

Experimental studies indicate that aging is accompanied by progressive impairment of intestinal barrier integrity, characterized by disruption of epithelial tight junctions, alterations in the mucus layer, and increased intestinal permeability. However, clinical evidence regarding intestinal barrier alterations during physiological aging in healthy humans remains scarce. To date, only a limited number of clinical studies have directly compared healthy young and older adults. One comprehensive study found no significant age-related differences in intestinal permeability or epithelial tight-junction integrity, suggesting that intestinal barrier dysfunction may become more apparent during pathological rather than physiological aging [65]. Notably, intestinal barrier aging may differ by sex. Recent human evidence suggests a more pronounced age-associated decline in intestinal barrier function in females, accompanied by increased circulating bacterial products and inflammatory markers [66].

Experimental evidence suggests that age-related barrier dysfunction is associated with gut microbial alterations, mucus layer thinning, and impaired epithelial homeostasis, which may facilitate the translocation of microbial products, toxins, and antigens and promote chronic low-grade inflammation [9,67,68]. Experimental studies consistently demonstrate age-dependent increases in intestinal permeability in rodents and non-human primates, accompanied by elevated circulating muramyl dipeptide and remodeling of tight junction proteins, including reduced expression of ZO-1, occludin, and JAM-A [7,68,69,70].

#### 4.1.2. Structural Remodeling of the Intestinal Barrier

Aging is accompanied by progressive structural remodeling of the intestinal epithelium, characterized by intestinal wall thinning, villus shortening, crypt remodeling, microvillus degeneration, and epithelial atrophy. Human morphometric studies have reported age-associated alterations in small-intestinal villous architecture, including shorter proximal jejunal villi in older individuals [71,72]. Consistent findings have been observed in multiple animal models, including mice, rats, and *Caenorhabditis elegans*, which exhibit progressive intestinal atrophy, luminal remodeling, shortened villi, crypt hyperplasia, and loss of microvilli during aging [7,35,73,74].

In addition to epithelial remodeling, aging also compromises the mucus barrier. Healthy individuals rely on an intact mucus layer to prevent direct microbial contact with the epithelium. During aging, mucus layer thickness is reduced owing to goblet cell loss and downregulation of mucin biosynthesis-related genes, accompanied by enrichment of mucus-degrading bacteria such as Bacteroides vulgatus. These alterations increase epithelial exposure to luminal microorganisms and contribute to impaired barrier function and greater susceptibility to intestinal inflammation [35,75,76]. These age-related barrier alterations also exhibit sex-specific features. In mice, aging affected intestinal mucus properties and barrier-related gene expression differently between males and females, with sex-dependent changes in mucus biosynthesis- and tight-junction-related genes [76].

Age-related remodeling also affects gut-associated lymphoid tissues. Both solitary intestinal lymphoid tissues (SILT) and Peyer’s patches undergo dynamic structural and cellular changes during aging. Although SILT structures increase in aged mice, Peyer’s patches exhibit altered immune cell composition, including increased CD4+ cells, reduced CD8+ cells, and decreased number and maturation of microfold (M) cells, suggesting impaired antigen sampling and mucosal immune surveillance [35,76,77,78].

Collectively, these structural alterations compromise epithelial renewal, mucus barrier integrity, and mucosal immune homeostasis, thereby increasing susceptibility to microbial invasion and chronic low-grade inflammation during aging.

#### 4.1.3. Changes in Intestinal Stem Cells

Preclinical studies indicate that aging can impair intestinal stem cell (ISC) function through both intrinsic cellular defects and niche remodeling. Intrinsic alterations, including cell cycle dysregulation, oxidative stress, and apoptosis, reduce ISC self-renewal and regenerative capacity, whereas activation of the mTORC1–PPAR-α–Notum pathway in aged Paneth cells inhibits Wnt signaling and further compromises ISC maintenance. In parallel, aging reshapes chromatin accessibility and differentiation programs, leading to impaired epithelial renewal, barrier dysfunction, and increased susceptibility to inflammation and tissue injury [79,80,81]. Single-cell analyses revealed that intestinal stem cell aging involves extensive transcriptional reprogramming of Lgr5^hi^ ISCs, disrupting Wnt signaling, cell-cycle control, and metabolic pathways. These changes delay progenitor maturation and compromise epithelial renewal, but can be partially reversed by gerotherapeutics, suggesting that ISC aging retains a degree of functional plasticity and therapeutic reversibility [82].

In addition, aging disrupts ISC signaling networks involved in maintaining stem cell homeostasis. Altered IFN-γ–ERK/MAPK signaling disturbs the balance between active and quiescent ISC states, leading to reduced stem cell pool preservation and impaired epithelial regeneration [83]. Aging-associated inflammatory activation and abnormal stress responses, including JNK, JAK/STAT, and EGFR pathway dysregulation, may further promote aberrant ISC proliferation and differentiation, ultimately disrupting intestinal epithelial organization and homeostasis [77,84].

Importantly, age-associated microbial dysbiosis may further exacerbate ISC dysfunction by promoting chronic inflammation, altering microbial metabolites, and disrupting epithelial–microbial interactions. However, current evidence is derived almost exclusively from animal models and in vitro studies, and clinical evidence validating age-related ISC dysfunction and ISC-targeted therapeutic strategies remains lacking.

### 4.2. Changes in Intestinal Function

#### 4.2.1. Immune Function

Aging is accompanied by a progressive decline in immune function, termed immunosenescence, characterized by alterations in both innate and adaptive immunity. Innate immune defects include impaired macrophage bactericidal activity, neutrophil chemotaxis, NK cell cytotoxicity, efferocytosis, and dendritic cell antigen presentation, along with reduced plasmacytoid dendritic cells (pDCs) and naïve B cells [7,9,27]. Adaptive immunity is also remodeled with aging, including memory T cell accumulation, Th1/Th2 imbalance toward Th17 polarization, and expansion of immunosuppressive regulatory T cells (Tregs), resulting in impaired immune regulation and increased disease susceptibility [9].

Cellular senescence further contributes to age-related immune dysfunction through the senescence-associated secretory phenotype (SASP), including cytokines and chemokines, thereby promoting chronic inflammation and contributing to multiple age-associated diseases [85]. Clearance of senescent cells has been shown to delay age-associated functional decline and extend lifespan, suggesting cellular senescence as a potential therapeutic target for healthy aging [86].

Age-related immune alterations also impair intestinal immune homeostasis. Aging reduces CD4^+^ T cell proliferation in Peyer’s patches, decreases immunoregulatory molecule expression in lamina propria CD4^+^ T cells, and increases Th17 cell abundance, leading to compromised mucosal immunity, gut microbiota imbalance, and weakened intestinal barrier function [7]. Sex-dependent immune aging has also been observed experimentally, with aged male and female mice showing distinct alterations in intestinal T-cell populations and cytokine profiles, indicating that mucosal immune remodeling during aging is not uniform between sexes [76,87].

Overall, immunosenescence is a key feature of intestinal aging. Declines in innate and adaptive immune function, together with SASP-associated inflammation, are closely linked to impaired intestinal immune homeostasis, gut microbial alterations, and systemic low-grade inflammation.

#### 4.2.2. Inflammaging

Aging is characterized by chronic low-grade inflammation, termed inflammaging, which contributes to the development of multiple age-related diseases [3]. Accumulating evidence indicates that immune and inflammatory pathways undergo substantial age-related changes [88].

Physiological aging is characterized by elevated circulating levels of pro-inflammatory mediators, including IL-1β, IL-6, TNF-α, IL-18, IL-1RA, and C-reactive protein (CRP), together with reduced IL-10 [9,27]. Persistent chronic inflammation prompts TNF-α to drive degradation of supportive components in vascular endothelial cells, leading to enhanced microvascular leakage in aged mice [89]. Elevated IL-6 compromises epithelial barrier integrity and regulates the balance between regulatory T (Treg) cells and pro-inflammatory Th17 cells. In contrast, the concentration of IL-8, an important mediator of early antimicrobial immunity, declines during aging [64].

Mechanistically, inflammaging is driven by persistent activation of multiple inflammatory pathways, including NF-κB signaling, NLRP3 inflammasome activation, and impaired autophagy. Age-related deterioration of autophagic function further enhances NF-κB activation, thereby establishing a sustained pro-inflammatory state [15]. Meanwhile, expression of trefoil factor 3 (TFF3), a secreted protein produced by intestinal goblet cells and a key mediator of intestinal epithelial protection and regeneration, progressively declines during aging and is further suppressed by TNF-α and IL-1β through NF-κB-dependent mechanisms [90,91]. Consistent with these findings, aged mice exhibit impaired bacterial clearance and elevated IL-6 and TNF levels compared with young controls [68]. Importantly, inflammaging may also exhibit sex-specific effects on intestinal homeostasis, with recent evidence implicating monocyte-associated inflammatory responses in the greater age-related impairment of intestinal barrier function observed in females [66].

Collectively, these findings identify inflammaging as a prominent feature of age-related intestinal dysfunction.

#### 4.2.3. Metabolic Function

Gut metabolic profiles undergo extensive remodeling during aging. Activities of digestive enzymes, including sucrase, isomaltase, lactase, and aminopeptidase, increase with age [92]. Cholesterol absorption is enhanced until middle age, whereas fatty acid β-oxidation declines because of reduced expression of transporters and enzymes such as CPT1A and CPT1B [7]. In addition, impaired L-arginine and 3-hydroxybutyrate metabolism is associated with disrupted gut homeostasis, cognitive decline, and reduced ISC proliferation [7,93]. Aging also decreases the abundance of enterocyte progenitors, absorptive colonocytes, goblet cells, enteroendocrine cells, and tuft cells in the proximal colon [7].

Age-related alterations in the gut microbiota are also associated with changes in microbial metabolic functions. At the functional level, predicted profiling identified age-associated differences in microbial pathways, with pathways related to acetyl-CoA biosynthesis, niacin degradation, and L-leucine catabolism showing enrichment, whereas sedrene biosynthesis, tRNA processing, and L-isoleucine biosynthesis were depleted [20]. Other pathways, including anaerobic energy metabolism, inositol catabolism, catechol degradation, and folate biosynthesis, have also been implicated in aging [94]. Sex-specific metabolic responses have also been observed in aged mice, in which aging-associated gut microbial alterations were accompanied by sex-dependent changes in systemic bile acid homeostasis and inflammation [95]. Targeted plasma metabolomic analysis identified age-associated differences in circulating metabolites related to lipid, nucleotide, fatty acid, and tryptophan metabolism, including increased levels of saturated long-chain fatty acids, stearic acid, cadaverine, and other aging-associated metabolites [96].

Several circulating molecules have also been proposed as biomarkers of aging. Serum L-methionine declines progressively with age, although methionine restriction paradoxically delays aging and extends lifespan in experimental models, highlighting the complex role of methionine metabolism in longevity [69,97]. In addition, mitochondrial stress-induced factors, including FGF21, GDF15, and humanin, increase with age and are associated with immune regulation, metabolic dysfunction and physical frailty [98]. Moreover, increased oxidative stress and ROS accumulation promote mitochondrial dysfunction and metabolic imbalance, as demonstrated by elevated ROS levels in aged *Drosophila* tissues [99].

Collectively, these findings demonstrate coordinated age-associated alterations in intestinal, microbial, and systemic metabolism, although their causal contribution to physiological decline remains incompletely defined.

#### 4.2.4. Other Molecular Alterations

Other molecular alterations also contribute to intestinal aging. The expression of pro-senescence genes, including *ANKRD4*, *CABP4*, and *DBNL*, increases progressively with age, whereas several anti-senescence genes involved in intestinal barrier protection, such as *CCT8* and *HMGCS2*, show age-related downregulation. In contrast, *CD82* expression increases during aging and may exert a protective effect against intestinal atrophy [7]. Telomere shortening represents another hallmark of aging, although the extent of telomere attrition varies among different tissues [100]. In addition, noncoding RNAs, particularly miRNAs, regulate aging-associated inflammatory responses and cellular homeostasis. Plasma miRNA profiling reveals age-dependent alterations, with reduced levels of miR-146a, miR-151a-3p, miR-181a-5p and miR-1248, while miR-126-3p increases in older individuals [101,102,103,104].

## 5. Interventions Targeting Gut Aging

Changes in the intestinal environment are closely linked to external factors such as diet and lifestyle, influencing immune regulation, inflammatory responses, and metabolic homeostasis. Therefore, targeting the gut microbiota has emerged as a potential strategy for modulating age-associated intestinal and systemic phenotypes. Current interventions mainly include FMT, dietary supplements, dietary modification, and other microbiome-based approaches, which aim to restore microbial balance and improve intestinal function.

### 5.1. Fecal Microbiota Transplantation (FMT)

#### 5.1.1. Human Evidence

Clinical studies investigating the effects of FMT on systemic aging or intestinal aging in older adults remain limited, with most evidence derived from animal models. Nevertheless, the potential of FMT as an intervention to promote healthy aging in humans has attracted increasing attention. A recently published double-blind, randomized, placebo-controlled clinical trial transplanted microbiota from young healthy donors into older participants using oral freeze-dried FMT capsules. The study aimed to comprehensively evaluate its effects on gut microbiota composition, inflammatory status, muscle function, physical performance, metabolic parameters, and cognitive outcomes [105]. Although clinical evidence remains limited, the rigorous design of this trial represents an important step toward translating preclinical FMT findings into potential interventions for promoting healthy aging.

Beyond aging-related interventions, clinical studies have also demonstrated the therapeutic potential of FMT in age-associated diseases. In patients with Parkinson’s disease, transplantation of fecal microbiota from healthy donors has been shown to induce long-term remodeling of gut microbial composition, increase the abundance of SCFA-producing bacteria, and improve motor symptoms, while maintaining a favorable safety profile [106,107].

#### 5.1.2. Animal/Mechanistic Evidence

FMT has demonstrated significant potential in reversing or improving age-related physiological changes. With respect to gut microbiome dynamics, FMT from aged mice to young hosts triggers the enrichment of aged-associated bacterial species: *Prevotella* sp. MGM2, *Lactobacillus intestinalis*, *Lachnospiraceae bacterium* 10–1, *Parabacteroides distasonis* and *Lactobacillus johnsonii.* Conversely, FMT from young mice to aged hosts promotes the colonization of youth-specific gut bacteria, such as *Muribaculaceae bacterium*, *Bifidobacterium animalis*, *Eubacterium* sp14–2 and *Akkermansia muciniphila* [108]. Similarly, pseudo-germ-free mice receiving young-donor microbiota exhibit microbial profiles enriched in genera such as *Ruminoclostridium* and *Lachnoclostridium*, whereas transplantation of aged-donor microbiota leads to an increased abundance of *Bacteroides*, resembling the microbial profile of aged donors [109]. In aged recipients, young-donor FMT increases microbial diversity and enriches several taxa, including *Akkermansia*, *Dubosiella*, *Alistipes*, and *Rikenellaceae*_RC9_gut_group [110]. Moreover, in the *Nothobranchius furzeri* model, transplantation of young microbiota alleviated age-related locomotor decline, restored microbial diversity, extended lifespan, and increased the abundance of youth-associated bacterial genera [111].

Beyond microbial composition, FMT exerts protective effects on intestinal homeostasis. Young-donor FMT effectively alleviates age-associated intestinal barrier dysfunction, as demonstrated by reduced circulating intestinal fatty acid-binding protein (I-FABP), a marker of epithelial injury. In aged mice receiving young microbiota, I-FABP levels were restored toward those observed in young controls, whereas young mice receiving aged microbiota exhibited increased intestinal damage markers [108]. In addition, young-donor FMT suppresses pro-inflammatory pathways, including NF-κB and IL-6 signaling, restores intestinal telomere function, and enhances tight junction integrity through upregulation of proteins such as occludin and claudin-1 [8,108,112]. Conversely, transplantation of aged-donor microbiota has been associated with reduced abundance of taxa linked to SCFA-related functions and with increased inflammatory responses, oxidative stress, and T-cell activation in experimental models [28,29,110,113,114].

The beneficial effects of FMT extend beyond the intestine. Young-donor microbiota transplantation has been shown to improve metabolic homeostasis, as evidenced by reduced insulin resistance, increased HDL levels, and decreased circulating ROS. It also enhances vascular endothelial function, attenuates vascular inflammation, delays aortic telomere shortening, improves immune dysfunction, promotes hippocampal neurogenesis, enhances cognitive performance, delays ovarian aging, and increases physical activity in aged animals [8,112,115,116,117,118]. Furthermore, the age of the microbiota donor may influence disease susceptibility. Mice receiving aged microbiota exhibited increased colon tumor incidence, elevated tumor burden, and enhanced inflammatory status compared with those receiving young microbiota [109].

Although some discrepancies exist among studies, young-donor FMT consistently demonstrates superior effects compared with aged-donor FMT in restoring intestinal barrier function and reducing inflammatory responses. Recent single-cell sequencing analyses further suggest that enhanced ATP synthesis and mitochondrial metabolic activity in intestinal epithelial cells may contribute to the cellular mechanisms underlying the anti-aging effects of FMT [119].

#### 5.1.3. Limitations and Translational Gaps

Animal studies have shown that FMT from young or healthy donors can reshape the gut microbiota of aged recipients, restore intestinal barrier integrity, reduce inflammation and metabolic disturbances, and improve aging-associated phenotypes, including frailty, muscle decline, immunosenescence, and cognitive impairment. However, current evidence remains largely limited to preclinical models, and clinical studies in naturally aging populations are still lacking. Future research should focus on evaluating the reproducibility of these benefits in humans, while addressing key challenges such as donor selection, safety evaluation, and long-term microbial engraftment stability. In addition, the development of next-generation probiotics and microbiota-targeted interventions may provide alternative strategies for improving age-related gut dysfunction.

### 5.2. Dietary Supplements

Probiotics and prebiotics are widely used microbiota-targeted dietary interventions. Probiotics contain live microorganisms that confer health benefits when administered in adequate amounts, whereas prebiotics are substrates selectively utilized by host microorganisms to confer health benefits. The following sections describe the intervention effects of these two common dietary supplements on intestinal aging.

#### 5.2.1. Probiotics

##### Human Evidence

In a randomized, double-blind, placebo-controlled clinical trial, van Zanten et al. demonstrated that multi-strain probiotic supplementation promoted the colonization of exogenous strains and improved several gastrointestinal symptoms in older adults. However, it did not significantly alter the overall gut microbiota composition or metabolic profiles, suggesting that the gut ecosystem in healthy older individuals exhibits considerable stability [120]. A randomized controlled trial further showed that probiotic supplementation with *Bifidobacterium bifidum* BGN4 and *Bifidobacterium longum* BORI produced sex-dependent changes in gut microbial composition and peripheral immune profiles in older adults. Probiotics reduced dendritic cells and CD14+CD16− monocytes in men, whereas these effects were not observed in women; conversely, total CD4+ T cells were reduced in women. Sex-specific changes were also observed in several inflammation-associated gut bacteria, suggesting that biological sex may influence microbiota and immune responses to probiotic supplementation [121]. Moreover, a 10-week intervention with a multi-strain probiotic combination containing *Lactobacillus rhamnosus* and *Bifidobacterium lactis* improved global cognitive function, memory performance, and emotional status in healthy older adults, providing preliminary clinical evidence that probiotic supplementation may influence selected neurocognitive outcomes [122].

##### Animal/Mechanistic Evidence

Animal studies further demonstrate that probiotics may alleviate age-related dysfunctions through multiple mechanisms, particularly by restoring intestinal homeostasis and reducing inflammatory alterations. *Lactobacillus fermentum* was shown to modulate aging-associated mTOR signaling in mouse preadipocytes, suppress SASP activation, and downregulate senescence-related markers, including p53 and p21 [123]. *Akkermansia muciniphila* enhances mucus layer function, restores goblet cell abundance, improves epithelial integrity, reduces intestinal permeability, and decreases circulating LPS levels, thereby promoting intestinal homeostasis and mitigating aging-related metabolic and inflammatory disorders [124,125,126]. *Lactobacillus plantarum* BFS1243 supplementation reversed frailty-associated intestinal alterations induced by FMT from frail elderly donors, including reduced butyrate production, impaired tight junction expression, decreased goblet cell numbers, and increased inflammation [127]. Collectively, these findings indicate that maintenance of intestinal barrier integrity represents one of the primary mechanisms underlying the anti-aging effects of probiotics.

Beyond intestinal effects, probiotics also exert systemic benefits by modulating inflammation, oxidative stress, and the gut–brain axis. *Phascolarctobacterium merdae* administration reduced frailty index, oxidative stress, and pro-inflammatory cytokine levels such as IL-6 and TNF-α in aged mice [128]. Furthermore, both viable and heat-inactivated *Lactobacillus paracasei* PS23 improved age-related cognitive deficits and anxiety-like behaviors, accompanied by enhanced hippocampal antioxidant capacity and increased intestinal butyrate levels [129]. Similarly, *Lactobacillus fermentum* PL9988 has demonstrated immunomodulatory, anti-inflammatory, and antioxidant properties, further supporting its potential as a probiotic candidate for promoting healthy aging [26]. *Roseburia inulinivorans* supplementation significantly improved grip strength in aged mice, promoted muscle fiber remodeling, and enhanced muscle energy metabolism, suggesting its potential as a next-generation probiotic candidate for maintaining physical function and mitigating age-associated frailty [130].

Emerging evidence also suggests that probiotics can directly regulate ISCs’ activity and cellular senescence. *Bifidobacterium adolescentis* ameliorated colonic senescence in mouse and organoid models by activating ISCs through Paneth-like cell-mediated Wnt/β-catenin signaling, accompanied by altered expression of aging-associated markers including p21, p53, and Lgr5 [131].

Compared with individual strains, multi-species probiotic formulations may exert broader anti-aging effects through functional complementation and synergistic interactions among different microbial species. In a *Drosophila* aging model, a bioactive probiotic consortium comprising *Lactobacillus plantarum* NCIMB 8826, *Lactobacillus fermentum* NCIMB 5221 and *Bifidobacterium longum* subsp. *infantis* NCIMB 702255 enhanced electron transport chain activity and upregulated SOD expression, thereby reducing age-associated oxidative stress and extending lifespan by over 50% relative to control animals [19]. Administration of the S7 bacterial consortium increased fecal acetate levels and reduced colonic permeability and plasma TNF-α concentrations in germ-free mice. Additionally, when S7 was administered to germ-free mice colonized with microbiota from a frail elderly donor, it predominantly increased the abundance of *Barnesiella intestinihominis*, showing potential to improve spatial reference memory and learning ability [132]. *Bifidobacterium bifidum* LTBB21J1, *Lactobacillus casei* LTL1361 and their combined formulation ameliorated anxiety-like behaviors and cognitive learning capacities in aged mice, and alleviated neuronal damage and oxidative-inflammatory lesions in the aging mouse brain [133]. A human-origin probiotic consortium, containing five *Lactobacillus* strains and five *Enterococcus* strains, increased taurine levels, fortified tight junction integrity, extended lifespan, attenuated obesity and reduced intestinal leakage [134]. Overall, accumulating evidence suggests that rationally designed probiotic consortia may provide broader therapeutic benefits than single-strain probiotics by simultaneously targeting multiple hallmarks of aging.

##### Limitations and Translational Gaps

Despite promising anti-aging effects observed in animal models, the translation of probiotic interventions into humans remains limited. Current challenges include the lack of long-term clinical evidence, strain-specific variability, inconsistent formulations, and individual differences in host microbiota. Moreover, most clinical studies focus on surrogate biomarkers rather than direct measures of biological aging, frailty, or longevity. The anti-aging effects of probiotic interventions reported in cellular, animal, and human studies are summarized in Table 3.

As summarized in Table 3, probiotic interventions have demonstrated beneficial effects on aging-related outcomes across cellular, animal, and human studies. Animal studies have reported improvements in lifespan, intestinal barrier function, cognitive performance, and inflammatory responses, whereas human studies have primarily demonstrated benefits in gastrointestinal health, immune regulation, and cognitive function. These effects may be associated with gut microbiota modulation, reduced oxidative stress and inflammation, and improved intestinal barrier integrity. However, the broader anti-aging effects observed in animal models have not yet been consistently reproduced in human studies. This discrepancy may reflect differences in probiotic strains, intervention duration and dosage, baseline microbiota, study populations, and outcome measures. Therefore, adequately powered and well-controlled clinical trials with standardized aging-related endpoints are needed to determine whether the benefits observed in preclinical models can be translated to healthy aging in humans.

#### 5.2.2. Prebiotics

##### Human Evidence

Recent randomized controlled trials have provided clinical evidence supporting the potential benefits of prebiotic supplementation in aging. In community-dwelling older adults, supplementation with an inulin and oligofructose-based prebiotic mixture improved frailty-related outcomes, including gait speed and grip strength, accompanied by favorable alterations in gut microbiota composition and microbial metabolism [138]. Similarly, a placebo-controlled trial in older adults with frailty syndrome demonstrated that supplementation with a prebiotic mixture based on inulin and fructooligosaccharides improved fatigue and handgrip strength, suggesting that prebiotic-mediated modulation of the gut microbiota–muscle–brain axis may help alleviate age-related functional decline [139].

##### Animal/Mechanistic Evidence

Specifically, in aged mice, galacto-oligosaccharides (GOS) alleviate age-induced increases in intestinal permeability, promote MUC2 expression and augment mucus thickness. Moreover, dietary GOS intervention substantially reduces the elevated TNF-α gene expression levels in the distal colonic tissues of aged mice [140].

Apart from standalone interventions, the integration of probiotics with bioactive phenolic formulations as prebiotics, also termed synbiotics, has shown effects on several aging-related phenotypes in experimental models. A prominent case in point is a synbiotic blend comprising *Triphala*, an *Ayurvedic* herbal extract abundant in polyphenols, and three probiotic strains: *Lactobacillus plantarum* Lp8826, *Lactobacillus fermentum* Lf5221 and *Bifidobacterium longum* subsp. *infantis* Bi702255. This synbiotic preparation showcased its longevity-promoting potential by significantly extending the lifespan of *Drosophila* and alleviating age-associated physiological stress, oxidative stress and low-grade inflammatory responses [19]. Another compelling study investigated the efficacy of agarooligosaccharides (AOS) in D-galactose-induced senescent mice. AOS intervention led to pronounced enhancements in overall murine health, manifesting as increased intestinal length, augmented *Muc2* expression, improved gut barrier function, restored ISC homeostasis and reduced inflammation levels. Fecal 16S rRNA sequencing further indicated that AOS increased gut microbial diversity and altered the relative abundance of specific microbial taxa. In particular, AOS increased the relative abundances of *Lachnospiraceae* NK4A136 and *Rikenellaceae*, accompanied by significantly increased fecal concentrations of acetate, propionate, butyrate, and isobutyrate [141].

##### Limitations and Translational Gaps

Current evidence is largely derived from animal models, while clinical studies directly evaluating intestinal barrier function, ISC homeostasis, and regenerative capacity in older adults remain lacking. Well-designed randomized clinical trials with mechanistic endpoints are needed to validate the translational potential of prebiotic interventions.

### 5.3. Diet

Dietary patterns are increasingly recognized as major modulators of aging, as different dietary habits can profoundly influence metabolic homeostasis, inflammation, and longevity. Current evidence has mainly focused on several representative dietary patterns, including the Mediterranean (MED) diet, ketogenic diet, high-fat diet, and high-sugar diet, which exhibit distinct and sometimes contrasting effects on aging-related outcomes.

Ketogenic diets can rebalance the gut microbiota, enhance cognitive function and repress neuroinflammatory marker expression; however, prolonged ketogenic interventions may also disrupt microbial diversity, increase intestinal inflammation, and impair barrier function [15]. The effects of high-fat and high-sugar diets on aging remain complex and context-dependent. For example, long-term high-fat feeding produces tissue-specific alterations in telomere length and telomerase activity in rodents, while glucose supplementation exhibits opposite effects depending on developmental stage and species [100,142,143]. The Mediterranean diet has been associated with favorable health and aging-related outcomes. However, evidence that it directly extends human lifespan or exerts these effects specifically through microbiota-mediated mechanisms remains limited. Polyphenols, as major bioactive components of the MED diet, regulate aging-related processes through multiple mechanisms, including inhibition of DNA methyltransferase 1, modulation of gut microbiota composition, enhancement of intestinal barrier integrity, and reduction in oxidative stress [144,145].

Among dietary polyphenols, anthocyanins exemplify the bidirectional interaction between dietary compounds and the gut microbiota [146]. Parent anthocyanins may modulate gut microbial composition, while unabsorbed anthocyanins can undergo microbial biotransformation into lower-molecular-weight phenolic metabolites. Human isotope-tracer studies have demonstrated extensive anthocyanin metabolism and systemic exposure to these metabolites [147], although evidence for brain exposure remains predominantly preclinical. In naturally aged mice, cyanidin-3-glucoside improved cognitive performance alongside alterations in gut microbial composition and SCFA-related metabolism and activation of the ERK/CREB/BDNF pathway [148]. Anthocyanin-related metabolites, including protocatechuic acid and 4-hydroxybenzoic acid, also showed metabolite- and context-dependent neuroprotective and anti-inflammatory effects in experimental models [149]. However, human evidence remains limited. A 24-week randomized controlled trial in adults aged 60–80 years found modest and heterogeneous microbiota responses to anthocyanin supplementation but no significant improvement in episodic memory or microbiota-mediated cognitive effects [150]. Similarly, another 24-week multicenter trial in adults aged 60–85 years found no significant cognitive benefit from either an anthocyanin-rich diet or anthocyanin supplementation [151]. Thus, although experimental evidence supports plausible microbiota–brain interactions, current clinical evidence does not establish a microbiota-mediated cognitive benefit of anthocyanins in older adults.

Tryptophan-rich diets may alleviate age-related dysfunction, whereas the abundance of bacteria involved in tryptophan and indole metabolism declines substantially with aging [16,29]. Similarly, age-related alterations in lipid metabolism, including reduced beneficial unsaturated fatty acids and increased detrimental saturated fatty acids, are closely associated with aging progression [96]. Preclinical studies suggest that dietary components such as taurine, ginsenosides, Panax ginseng, and theaflavins may influence intestinal homeostasis and aging-related pathways. However, their effects vary by compound and experimental context, and their efficacy in delaying human aging remains to be established [152,153,154,155,156]. *Eucommia ulmoides* polysaccharides and caffeic acid delay the decline of intestinal function in aged *Drosophila* by reducing oxidative stress, suppressing the growth of *Gluconobacter*, *Providencia*, and *Enterobacteriaceae* in senescent flies, and ultimately extending their lifespan [99,157]. Dietary fiber extends *Drosophila* lifespan by upregulating Duox expression in the gut, increasing the abundance of *Asaia* and *Alloprevotella* in the gut microbiome, and significantly decreasing the levels of *Bacillus*, *Pseudomonas*, *Listeria*, and *Enterococcus* [158]. Branched-chain amino acids, including leucine, isoleucine, and valine, regulate TOR signaling and contribute to metabolic health and longevity [159,160].

*Oudemansiella raphanipes* polysaccharide alleviates age-associated gut dysbiosis by promoting the proliferation and adhesion of *Lactobacillus acidophilus* and *Bifidobacterium bifidum* and strengthens intestinal barrier integrity through regulation of apoptosis and tight-junction proteins [161]. In aged-mouse models, *Astragalus* polysaccharides and *Astragalus* ethanol extract have been reported to improve intestinal barrier integrity and selected aging-related outcomes, potentially involving changes in *Lactobacillus* abundance and IL-22 signaling-mediated intestinal stem cell regeneration [162,163]. Notably, certain food-derived bioactive compounds, such as rapeseed peptide Maillard reaction products, may contribute to healthy aging by simultaneously enhancing food properties and reducing oxidative stress, inflammation, and gut microbial dysbiosis [164].

### 5.4. Other Interventions

Modifications to daily lifestyle can also elicit beneficial regulatory effects on gut microbiota, such as consistent physical activity and caloric restriction (CR) [9]. CR can trigger inhibition of insulin-like growth factor-1 (IGF-1) and insulin signaling, rebalance gut microbial composition and promote the proliferation of *Lactobacillus*-predominant gut microbiota, thereby enhancing host health and promoting longevity [15,29,165]. Intermittent fasting (IF) alleviates age-related intestinal remodeling, strengthens intestinal barrier function, and extends the lifespan of *Drosophila* [164]. Dietary restriction (DR) augments enterocyte resilience by upregulating dMyc gene expression in the intestinal epithelium, inhibits the increase in intestinal permeability and the dysregulated proliferation of ISCs, and thus supports the maintenance of intestinal homeostasis [166].

Nicotinamide mononucleotide (NMN) has emerged as a potential intervention for age-related functional decline. Growing evidence suggests that its beneficial effects are accompanied by alterations in the gut microbiota and host metabolism. Short-term NMN supplementation in pre-aging mice significantly altered fecal microbial composition, including altered abundances of *Helicobacter* and *Desulfovibrio*, accompanied by increased telomere length [167]. More recently, long-term NMN treatment remodeled the gut microbiota in ageing mice, including increased abundance of *Bifidobacterium pseudolongum* and *Akkermansia muciniphila*, alongside improved intestinal barrier integrity, tight junction protein expression, and intestinal stem cell activity. In parallel, NMN prolonged lifespan and delayed senescence in prematurely aged mice [168].

Pharmacological elimination of senescent cells provides another avenue for targeting aging-associated intestinal dysfunction. Treatment with the senolytic combination dasatinib and quercetin reduces intestinal senescent cell accumulation, decreases p16 and p21 expression, preserves villus and crypt structures, and restores gut microbial balance in aged mice [169,170]. In experimental models, dietary supplementation with α-ketoglutarate (AKG) extends lifespan, alleviates inflammatory and frailty phenotypes, and enhances butyrate-producing metabolic pathways [18].

Although microbiota-targeted interventions have shown effects on multiple aging-related phenotypes, particularly in experimental models, their translational potential varies substantially according to the available level of evidence. Table 4 summarizes the current evidence supporting different microbiota-targeted strategies from human clinical trials and animal/mechanistic investigations.

Overall, microbiota-targeted interventions have demonstrated promising effects on aging-related phenotypes, particularly in preclinical models. However, the strength of clinical evidence varies across intervention strategies. While probiotics and prebiotics have shown potential benefits for selected gastrointestinal, immune, and physical outcomes in older adults, direct evidence supporting their efficacy in delaying intestinal aging remains limited. Similarly, although FMT and certain dietary interventions have demonstrated beneficial effects in experimental studies, their clinical efficacy in promoting healthy aging has not been firmly established. These findings highlight a substantial gap between preclinical evidence and clinical translation, emphasizing the need for well-designed, large-scale, long-term randomized controlled trials with standardized aging-related outcomes.

## 6. Conclusions

Aging is accompanied by substantial alterations in gut microbial composition and function, which are increasingly associated with age-related physiological decline and chronic diseases. Although accumulating evidence has highlighted the involvement of the gut microbiota in aging, substantial heterogeneity across populations and studies complicates the identification of consistent microbial signatures, and the underlying mechanisms and translational potential of microbiota-targeted interventions remain incompletely understood.

In this review, we summarized the relationship between gut microbiota and aging from three perspectives. First, we summarized age-associated alterations in gut microbial composition and functional profiles, highlighting both recurring microbial features and substantial population- and condition-specific variation. Second, we examined evidence linking age-associated microbial alterations with intestinal barrier dysfunction, impaired epithelial homeostasis, immune dysregulation, and chronic low-grade inflammation, while distinguishing human associations from mechanistic evidence derived primarily from experimental models. Third, we reviewed emerging microbiota-targeted strategies, including FMT, probiotics, prebiotics, and dietary interventions, while emphasizing their current evidence levels and translational challenges.

**Figure 1:**
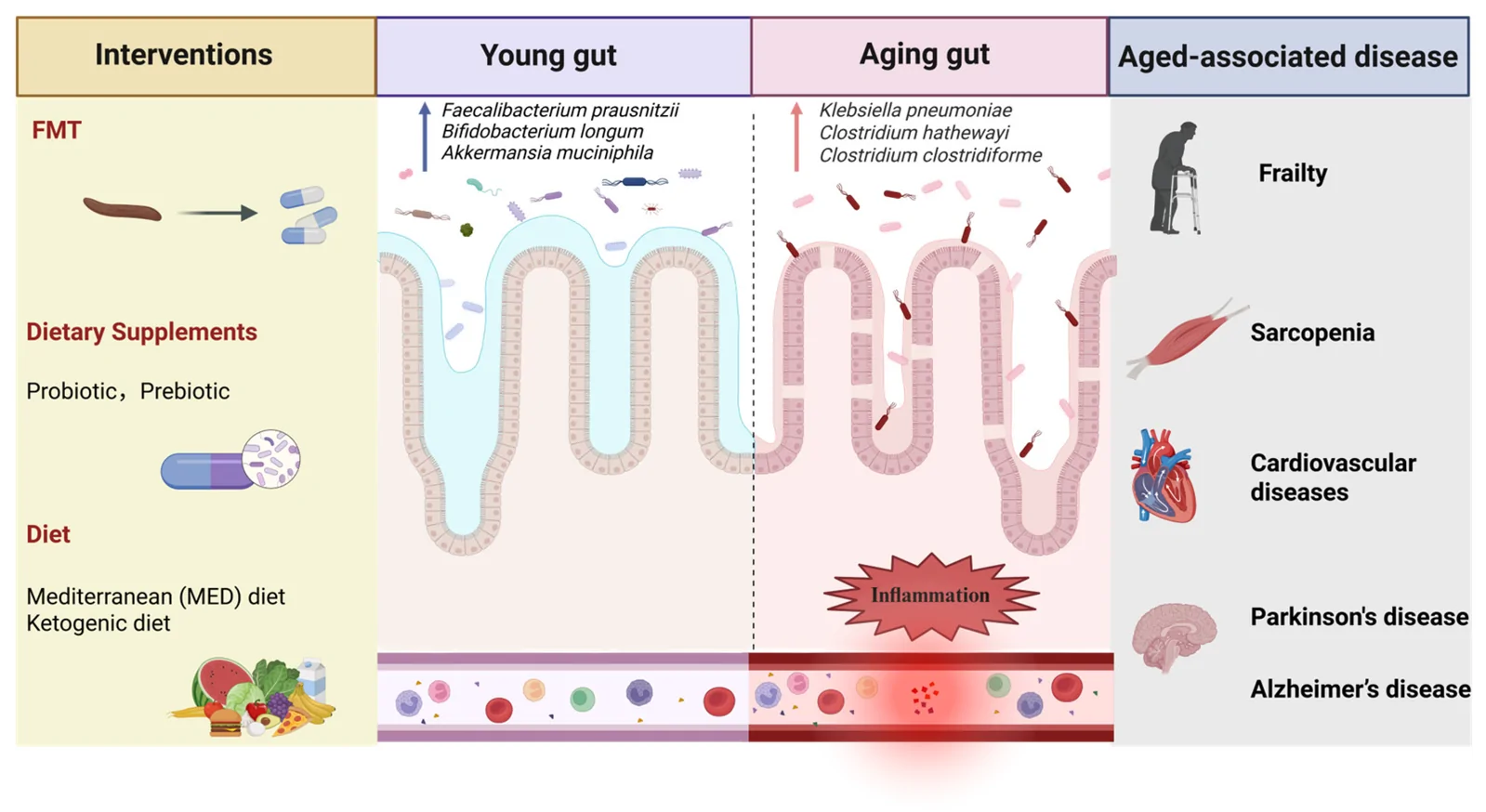
Gut microbiota remodeling during aging and potential microbiota-targeted interventions for healthy aging. Aging is associated with alterations in gut microbial composition and function, intestinal barrier integrity, and inflammatory activity. These interconnected features have been associated with age-related conditions, including frailty, sarcopenia, cardiovascular diseases, Parkinson’s disease, and Alzheimer’s disease. Their relationships are likely bidirectional and may be influenced by host, environmental, dietary, medication-related, and disease-associated factors. Microbiota-targeted strategies, including FMT, probiotics, prebiotics, and dietary interventions, may modulate the gut microbial ecosystem and intestinal homeostasis; however, evidence for their efficacy in delaying human aging remains limited.

As summarized in Figure 1, aging is associated with remodeling of the gut microbiota, accompanied by alterations in intestinal barrier integrity and increased inflammatory activity. These interconnected changes are associated with multiple age-associated conditions, including frailty, sarcopenia, cardiovascular diseases, Parkinson’s disease, and Alzheimer’s disease. Microbiota-targeted strategies, including FMT, probiotics, prebiotics, and dietary interventions, may modulate the gut microbial ecosystem and intestinal homeostasis, although their clinical efficacy and long-term benefits in healthy aging remain to be established.

## 7. Future Perspectives

Despite growing interest in the role of the gut microbiota in aging, it remains difficult to distinguish microbial changes associated with biological aging from those related to environmental exposures and disease status. Dietary patterns, malnutrition, medication use, prolonged hospitalization, physical inactivity, and chronic diseases can all affect gut microbial composition and function. As these factors become more common with age, their effects may overlap with those of the aging process itself. Similar microbial alterations may also occur in younger individuals exposed to comparable environmental or clinical conditions. However, overlapping microbial profiles do not necessarily imply equivalent disease phenotypes or indicate that these alterations are specific to biological aging. Moreover, the relationship between the gut microbiota and age-related physiological changes is likely bidirectional. Microbial alterations may affect host physiology, while changes in gastrointestinal function, immune status, dietary habits, medication use, frailty, and disease burden may further reshape the microbial community. These interrelated influences complicate the interpretation of age-associated microbial patterns, particularly in cross-sectional studies.

Although animal models have provided valuable insights into the potential roles of the gut microbiota in intestinal barrier integrity, inflammation, immune aging, and lifespan regulation, evidence from human studies remains predominantly observational. Longitudinal cohort studies with repeated microbiome assessments are needed to characterize microbial changes over time and establish their temporal relationships with aging-related outcomes. Comparisons between younger and older individuals with similar environmental and clinical exposure profiles, together with appropriate control for potential confounding factors, may help clarify the extent to which observed microbial changes are associated with aging rather than cumulative exposures or disease status. Integrating microbial profiles with functional and metabolomic data may further improve the identification of changes associated with biological aging. Well-designed intervention studies are also needed to investigate whether specific microbial alterations contribute to age-related physiological decline. Well-designed intervention studies are also needed to investigate whether specific microbial alterations contribute to age-related physiological decline.

Methodological heterogeneity represents another challenge in the interpretation of current findings. Differences in sequencing strategies, including 16S rRNA profiling and metagenomic sequencing, as well as variations in sample collection, processing procedures, and analytical pipelines, have limited direct comparisons between studies. Future research should incorporate standardized methodologies and integrate multi-omics approaches, including metagenomics, metabolomics, proteomics, and single-cell technologies, to further elucidate microbiota–host interaction networks and the mechanisms underlying intestinal aging.

Future studies on intestinal aging should move beyond descriptive characterization toward more accurate prediction and targeted intervention. Integrating microbial, metabolic, immune, and host-related information may provide a more comprehensive understanding of the complex interactions between gut microbiota and aging processes. Overall, gut microbiota is not merely a marker of aging but may serve as an important regulatory target for promoting healthy longevity. With advances in longitudinal studies, mechanistic investigations, and clinical trials, microbiome-based precision strategies may offer new opportunities for delaying age-associated decline and improving health outcomes in the growing elderly population.

## Figures and Tables

**Table 2:** Representative gut microbial alterations and functional evidence across selected age-associated conditions.

Conditions	Reported Microbial Alterations	Functional or Metabolite-Related Findings	Evidence Type	Consistency and Limitations
Frailty [36,37,38,39]	↑ Genera: *Parabacteroides*, *Veillonella*, *Alistipes* and *Klebsiella* ↓ Genera: *Prevotella*, *Faecalibacterium*, *Roseburia*	Associations with SCFA-related microbial functions, appetite regulation, physical performance, and muscle metabolism	Human observational studies and meta-analyses.Experimental animal evidence	Consistent decreases in *Prevotella*, *Faecalibacterium*, and *Roseburia* were reported across multiple studies, whereas most other taxonomic associations were cohort-specific; evidence for differences in α-diversity remains inconsistent.
Sarcopenia [40,41,42,43,44]	↑ Genera: *Escherichia-Shigella*, *Enterobacter*, *Streptococcus*; Species: *Desulfovibrio piger*, *Clostridium symbiosum*, *Hungatella effluvii* ↓ Genera: *Faecalibacterium*, *Roseburia*, *Blautia*, *Bifidobacterium*, *Lactobacillus*, *Prevotella*	Shotgun metagenomic functional profiling showed depletion of phenylalanine, tyrosine, and tryptophan biosynthesis and enrichment of α-linolenic acid metabolism, furfural degradation, and staurosporine biosynthesis.	Human observational studies and meta-analyses.Experimental animal evidence	β-diversity differences have been reported, whereas findings for α-diversity and specific taxa remain heterogeneous across studies; most human evidence is observational.
Cardiovascular diseases [45,46,47,48]	↑ Phylum: Proteobacteria; Genus: *Streptococcus* ↓ Genus: *Faecalibacterium*	Elevated circulating TMAO in CVD is associated with impaired endothelial function during aging. Microbial cholesterol, γ-butyrobetaine, and flavonoid metabolism are associated with cardiovascular health.	Human observational studies and meta-analyses.Experimental animal evidence	TMAO acts as a metabolic mediator linking gut dysbiosis with vascular aging
Parkinson’s disease [49,50,51,52]	↑ Family: Enterobacteriaceae; Genera: *Akkermansia*, *Desulfovibrio* ↓ Families: Lachnospiraceae, Prevotellaceae; Genera: *Prevotella*, *Faecalibacterium*, *Roseburia*	Fecal acetate, propionate, and butyrate were decreased, whereas circulating propionate, butyrate, and valerate were increased in PD.	Human observational studies and meta-analyses.Experimental animal evidence	Substantial between-cohort heterogeneity remains. SCFA concentration alterations are supported by direct metabolite measurements, whereas metagenomic pathway alterations represent functional potential.
Alzheimer’s disease [53,54,55,56]	↑ Phylum: Proteobacteria; Genera: *Escherichia/Shigella*, *Alistipes*, *Bacteroides*, *Bifidobacterium* ↓ Genera: *Faecalibacterium*, *Roseburia*, *Ruminococcus*, *Eubacterium*	Alterations in microbial gene families involved in carbohydrate, amino-acid, energy, glycan, and vitamin metabolism. Microbial compositional and functional features were associated with CSF biomarkers of AD and related pathologies. Microbiota-associated inflammatory signaling was linked to Aβ deposition, Tau hyperphosphorylation, oxidative stress, and microglial activation.	Human observational studies and meta-analyses.Experimental animal evidence	Microbial alterations vary across AD stages and cohorts. Current human evidence supports associations between microbial compositional/functional features and AD pathology, but does not establish causality.

Abbreviations: ↑, higher relative abundance; ↓, lower relative abundance; CVD, cardiovascular disease; SCFA, short-chain fatty acid; BCAA, branched-chain amino acid; TMAO, trimethylamine N-oxide; Aβ, amyloid-β.

**Table 3:** Effects of Probiotic Interventions on Aging-Related Outcomes in Cellular, Animal, and Human Studies.

Probiotic	Intervention Details	Effect	Model
*Bifidobacterium animalis* subsp. *lactis* Bi-07, *Bifidobacterium animalis* subsp. *lactis* Bl-04, *Lactobacillus acidophilus* NCFM, and *Lactobacillus paracasei* Lpc-37	Dose: 1 × 10^10^ CFU Duration: 24 weeks, daily; Age: 75 years; n = 98	Improved gastrointestinal symptoms in older adults [120].	Human
*Bifidobacterium bifidum* BGN4 and *Bifidobacterium longum* BORI	Dose: 1 × 10^9^ CFU; Duration: 12 weeks, daily; Age: >65 years; n = 53	Reduced the abundance of certain inflammation-associated bacteria and modulated peripheral immune cell profiles, including CD14^+^CD16^−^ monocyte proportions and CD4^+^ T cell proportions [121].	Human
*Lactobacillus rhamnosus* and *Bifidobacterium lactis*	Dose: 3.3 × 10^9^ CFU; Duration: 10 weeks, daily; Age: >55 years; n = 33	Improved cognitive function, memory performance, and emotional status [122].	Human
*Lactobacillus fermentum*	Concentration: 5% and 10% (*v*/*v*) probiotic-conditioned medium; Treatment duration: 3 days.	Reduction in PI3K/Akt/mTOR phosphorylation and decreased expression of aging markers (p53, p21WAF1, SA-β-gal, p38MAPK, iNOS, cox-2, ROS, NF-κB) [123].	3T3-L1 cells
*Akkermansia muciniphila*	Dose: 100 μL/mouse (CFU not reported); Duration: From 12 weeks to death, three times a week; Age: 12 weeks to death; n = 9	Extended lifespan, improved gut health (restoration of goblet cell numbers, thickened mucus layer, enhanced barrier function, reduced LPS levels) [126].	Mice
*Lactobacillus plantarum* BFS1243	Dose: 1 × 10^8^ CFU; Duration: 9 weeks; Age: 18–20 weeks; n = 18	Repair aging-related damage to the intestinal barrier, reduce inflammatory factors, and enhance physical activity capacity [127].	Mice
*Phascolarctobacterium merdae*	Dose: 4 × 10^7^ CFU Duration: 6 weeks, daily; Age: 8 weeks; n = 5	Reduced the FI aging score, oxidative stress levels, and inflammatory factors IL-6 and TNF-α [128].	Mice
*Lactobacillus paracasei* PS23	Dose: 1 × 10^9^ CFU; Duration: 9 weeks, daily; Age: 8 weeks, treated by D-galactose; n = 6	Improved spatial memory deficits and anxiety-like behaviors, enhanced antioxidant capacity, increased Lactobacillus and Bifidobacterium species, and elevated butyrate levels [129].	Mice
*Roseburia inulinivorans*	Dose: 4 × 10^7^ CFU; Duration: 8 weeks, three times per week; Age: 6 weeks; n = 8	Improve grip strength in aged mice, promoted muscle fiber remodeling, and enhanced muscle energy metabolism [130].	Mice
*Bifidobacterium adolescentis*	Dose: 1 × 10^9^ CFU; Duration: 4 weeks, every other day; Age: 12 months; n = 6	Activation of ISCs via the Paneth-like cell -mediated Wnt/β-catenin signaling pathway improves colonic aging [131].	Mice
S7 (*Alistipes putredinis*, *Barnesiella intestinihominis*, *Coprococcus catus*, *Dorea longicatena*, *Eubacterium rectale*, *Faecalibacterium prausnitzii*, and *Roseburia hominis*)	Dose: 2.77 × 10^8^ CFU; Duration: 4 weeks, once weekly; Age: Germ-free mice colonized with gut microbiota from elderly human donors; n = 10	Increased acetic acid concentration, enhanced intestinal barrier function, reduced inflammation, and improved cognitive function [132].	Mice
Five *Lactobacillus* strains (*L. paracasei* D3-5, *L. rhamnosus* D4-4, *L. plantarum* D6-2, *L. rhamnosus* D7-5, *L. plantarum* D13-4) and five *Enterococcus strains* (*E. raffinosus* D24-1, *E. INBio* D24-2, *E. avium* D25-1, *E. avium* D25-2, *E. avium* D26-1)	Dose: 1 × 10^9^ CFU/mL; Duration: 10 weeks, daily; Age: 80 weeks; n = 8	Increase taurine intake to strengthen tight junctions [134].	Mice
*Bifidobacterium bifidum* P61 and *Lactobacillus paracasei* P62	Dose: 1 × 10^9^ CFU; Duration: 8 weeks, daily; Age: 18 months; n = 6	By modulating AKT, NF-κB, and FOXO3a signaling pathways, it alleviates sarcopenia and cognitive impairment in aged mice [135].	Mice
*Bifidobacterium bifidum* BGN4 and *Bifidobacterium longum* BORI	Dose: 1 × 10^9^ CFU; Duration: 30 days, daily; Age: 15 months; n = 5	Improve age-related changes in neurons; enhance cognitive and motor functions [136].	Mice
*Lactobacillus fermentum* LTP1332 and *Bacteroides fragilis* LTBF12	Dose: 2 × 10^7^ CFU/day; Duration: 6 weeks, daily; Age: 20 months; n = 12	Reduced anxiety in mice, decreased neuroinflammation, lowered oxidative stress markers, and altered longevity-associated microbiota [137].	Mice
*Lactobacillus plantarum* NCIMB 8826, *Lactobacillus fermentum* NCIMB 5221 and *Bifidobacterium longum* subsp. *Infantis* NCIMB 702255	Dose: 3 × 10^9^ CFU/mL; Duration: 30 days; Age: Newly eclosed adults; n = 5 independent groups of 25 flies	Extend lifespan and reduce oxidative stress [19].	*Drosophila*

Abbreviations: CFU, colony-forming units; FI, Frailty Index; SA-β-gal, senescence-associated β-galactosidase; IL-6, interleukin-6; TNF-α, tumor necrosis factor α; ROS, reactive oxygen species; NF-κB, nuclear factor-κB.

**Table 4:** Clinical and preclinical evidence for microbiota-targeted interventions in gut aging.

Intervention	Human Clinical Trial Evidence	Animal/Mechanistic Evidence
FMT	Limited. Although randomized controlled trials of FMT in older adults have been initiated and clinical studies have been conducted in patients with Parkinson’s disease, direct evidence supporting its benefits for healthy aging remains insufficient [105,106,107].	Strong. Young-to-aged microbiota transfer improves metabolic function, inflammation, and aging-related phenotypes in animal models [8,108,112].
Probiotics	Moderate Clinical trials have reported improvements in gastrointestinal symptoms, immune function, and cognitive performance in older adults, although direct evidence for delaying aging remains limited [120,121,122].	Strong. Multiple probiotic strains have been shown to improve intestinal barrier integrity, modulate immune responses, and attenuate inflammation in aged animal models [123,124,125,126,127].
Prebiotics	Moderate. Randomized controlled trials have reported improvements in physical performance and gut microbiota composition in older adults, although direct evidence for delaying intestinal aging remains limited [138,139].	Strong. Prebiotic supplementation improves SCFA fecal concentrations and intestinal homeostasis in aged animals [140,141]
Diet	Limited. Long-term controlled trials specifically targeting intestinal aging are lacking	Strong. Dietary interventions modulate microbiota composition, metabolites, and inflammatory pathways in animal models [144,145,161,162,163]

Note: The strength of evidence was qualitatively classified into three levels: Limited, based on a small number of studies or preliminary findings with insufficient validation; Moderate, supported by multiple studies with generally consistent findings but some methodological limitations; and Strong, supported by multiple independent studies with consistent and reproducible findings. These classifications were based on the quantity, consistency, and methodological quality of the available evidence and were assessed separately for human clinical trials and animal/mechanistic studies. The ratings represent a qualitative narrative assessment rather than formal systematic evidence grading.

## Data Availability

No new data were created or analyzed in this study. All data used in this review are from previously published studies, which are cited in the article.

## Abbreviations

The following abbreviations are used in this manuscript:

IBD	Inflammatory bowel disease
FMT	Fecal microbiota transplantation
SCFAs	Short-chain fatty acids
ISCs	Intestinal stem cells
LPS	Lipopolysaccharide
TMAO	Trimethylamine N-oxide
PD	Parkinson’s disease
AD	Alzheimer’s disease
ROS	Reactive oxygen species
MED	Mediterranean
GOS	Galacto-oligosaccharides
BCAA	Branched-chain amino acid
CVD	Cardiovascular disease
